# A qualitative interview study on psycho-oncologists’ experiences with patient deaths in Germany

**DOI:** 10.1038/s41598-025-06991-x

**Published:** 2025-07-01

**Authors:** Svenja Wandke, Lisa Brandes, Mareike Rutenkröger, Hannah Führes, Klaus Lang, Martin Härter, Karin Oechsle, Carsten Bokemeyer, Isabelle Scholl

**Affiliations:** 1 Department of Medical Psychology, Center for Psychosocial Medicine, University Medical Center Hamburg-Eppendorf, Hamburg, Germany; 2 II. Department of Medicine, University Medical Center Hamburg-Eppendorf, Hamburg, Germany; 3 Psychotherapeutic Practice, Munich, Germany

**Keywords:** Cancer care, Patient death, Professional grief, Psycho-oncology, Qualitative interview study, Cancer, Psychology, Health care

## Abstract

Despite regular exposure to patient deaths, little research has explored how psycho-oncologists experience and cope with these events. This study examined the unique challenges faced by psycho-oncologists in Germany, aiming to contribute to the broader understanding of professional grief and its impact on healthcare professionals’ mental health and well-being. We conducted 25 semi-structured interviews with psycho-oncologists. The interviews were audiorecorded, transcribed, and analyzed using content analysis. Participants experienced an average of two patient deaths per month. The findings revealed both positive and negative effects of patient deaths on their personal and professional lives. While many participants managed professional grief successfully, most expressed unmet needs for education and information on coping strategies. Peer support was identified as particularly valuable for managing the emotional toll. Overall, patient deaths had significant, varying impacts on psycho-oncologists, highlighting the need for healthcare institutions to implement targeted support and educational interventions to address professional grief and enhance their well-being.

**Supplementary Information:**

The online version contains supplementary material available at 10.1038/s41598-025-06991-x.

## Introduction

Cancer remains one of the leading causes of death worldwide^1^ making patient loss a frequent occurrence for healthcare professionals (HCPs) in cancer care^2^. Previous to their patient’s death HCPs may still form close bonds with their patients due to the intensive and sometimes long-term nature of the care they provide^3^. Typically, grief follows the loss of someone with whom a close bond was shared^4^. However, traditional views of grief focus on personal relationships rather than the professional ones shared between HCPs and their patients^5^. Evidence suggests that professional grieving experiences are a unique experience distinct from personal grief, although the distinction between the two is not always clear^5^. In a recent scoping review, we reported relevant evidence to distinguish these phenomena^6^. The experience of professional grief has been separated from grief in general by the relatively high frequency of encounters with patient deaths, however often being less intense and confined to the workplace. Professional grief can include feelings of responsibility or guilt, which, unlike personal grief, might be linked to the professional role, sometimes leading HCPs to perceive their reactions as socially unacceptable or inappropriate^6^.

The impact of patient deaths on HCPs’ mental health and well-being is significant. Evidence indicates that professional grief can have detrimental effects on HCPs, affecting their mental health^7^overall well-being and quality of life^3,5,7,8^ as well as the quality of care they provide^9,10^. Despite the critical importance of understanding these experiences, there is a scarcity of research on how psycho-oncologists, especially within European healthcare contexts, navigate professional grief^5^, develop coping mechanisms, and how successful coping may be supported by healthcare institutions.

Given this gap, our study aimed to explore the experiences and coping strategies of psycho-oncologists in Germany regarding the death of cancer patients under their care. By focusing on this specific group, we seek to shed light on the unique challenges they face and contribute to the broader understanding of professional grief and its implications for HCPs’ mental health and well-being.

## Methods

### Study design

We conducted a qualitative interview study guided by criteria for reporting qualitative research (COREQ)^11^. The COREQ checklist can be found in supplementary file 1. This study was carried out according to the latest version of the Helsinki Declaration of the World Medical Association and respecting principles of good scientific practice. The local Ethics Committee of the University Medical Center Hamburg-Eppendorf gave approval prior to investigation (approval number: LPEK-0614). Study participation was voluntary and no foreseeable risks for participants resulted from the participation in this study. Participants were fully informed about the aims of the study, data collection and the use of collected data and written informed consent was obtained.

To ensure data security and confidentiality, audio recordings were deleted after transcription. All data, including transcripts and recordings prior to deletion, were securely stored within a password-protected IT environment (KIS2) at the University Medical Center Hamburg-Eppendorf (UKE), accessible only to members of the research team. No data were transmitted externally; all team members accessed data directly within the secure environment. In accordance with local regulations, transcripts will be stored for 10 years. Data analysis was conducted in German, with only selected transcript excerpts translated for publication.

### Sampling, recruitment and data collection

Participants were recruited via relevant professional associations, namely the Psycho-Oncology Working Group of the German Cancer Aid’s Network of Comprehensive Cancer Centers (Arbeitsgruppe Psychoonkologie des *Netzwerks Onkologische Spitzenzentren - CCC*), the State Consortium for Cancer Counselling Services (*Bundesarbeitsgemeinschaft für Krebsberatungsstellen e.V.)*, the Association for Continued Education in Psycho-Social Oncology *(Weiterbildung Psychosoziale Onkologie e.V.)*, the German Cancer Association’s Consortium for Psycho-Oncology (*Arbeitsgemeinschaft Psychosoziale Onkologie der Deutschen Krebsgesellschaft*) and a regional working group of psycho-oncologists based in the metropolitan area of Hamburg (*Psychoonkologie Treffen – POT*). The named associations forwarded our invitation for participation in the study to all their members via email or newsletter. Interested individuals contacted the first author, who provided detailed information about the study. Upon obtaining informed consent, individual interviews were scheduled. Notably, there were no dropouts after initial contact.

Participants were required to be professionally involved in psycho-oncological care, specifically providing psychosocial support to cancer patients, and to have experienced at least one patient death. Additionally, they needed sufficient proficiency in German to participate in the interview. No other inclusion or exclusion criteria were specified.

The selection process began on June 21, 2024, with the first invitation, and concluded on February 1, 2025, with the final inclusion. In total, 47 candidates expressed interest in the study, of whom 25 were interviewed.

The estimated sample size was based on achieving pragmatic saturation, informed by our prior experience with this methodology and sample sizes of other qualitative studies in this field of research^6,12^. We anticipated pragmatic saturation with around 25 interviews. We adopted a maximum-variation approach to include participants with varying ages, years of experience as psycho-oncologists, specific employment situations (e.g., inpatient or outpatient care), and regional locations (e.g., different federal states).

The first author (SW), a female psycho-oncologist with experience in psychological research and interviewing, conducted one-time individual semi-structured telephone interviews in German between November 2023 and February 2024. All interviews were audio-recorded. At the outset, participants were asked to recall a patient death of their choosing and, if willing, to share details about the case. Subsequently, they answered questions about their experiences with patient deaths, their coping, and current needs. The interview questions were derived from extensive qualitative research on HCPs’ experiences and coping with cancer patient deaths^6,13–18^. The complete interview guide is available in supplementary file 2. No field notes were taken, and we achieved our previously estimated goal of conducting 25 interviews. After the interview, participants completed a short paper-pencil questionnaire regarding their demographic and work-setting characteristics, returning it via mail.

SW conducted all interviews from a private office at the University Medical Center Hamburg-Eppendorf, while participants were asked to ensure they were as well in a quiet and private location during the interviews. SW had no prior close relationship with most participants; she only knew three participants from brief interactions at conferences and meetings. All participants were informed that the study was part of SW’s PhD research.

A pilot interview was conducted with a psycho-oncologist from the same inpatient clinic as the first author. This pilot interview was not included in the study results.

### Transcription and data analysis

The audio recordings were transcribed verbatim and imported into MAXQDA^19^ for analysis. Transcription followed a two-step process: initially, a preliminary transcript was generated using WhisperSpeaker^20^ a large-language model-based program, which combines several open source libraries, including Whisper from OpenAI for speech recognition and pyannote.audio for speaker diarization. This transcript was then manually reviewed and corrected by SW and LB (co-author, psychologist, medical student and research assistant) while listening to the original recordings. Transcripts were anonymized by removing all identifiable information (e.g., names, workplaces).

We employed inductive content analysis^21^ adhering to a detailed, pre-agreed analysis plan. SW and LB independently coded the first eight interviews to develop a code structure, which was then reviewed and finalized by the research team (SW, LB, MR, IS). SW coded the remaining interviews, with LB revising the codes. Discrepancies were resolved through discussion. The evolving code structure was reviewed and agreed upon by the research team after every eight interviews. Data analysis was conducted in chronological order of all interviews and began while interviewing the last two participants, thereby taking place partly during data collection. Transcripts and results were not returned to participants for feedback.

### Findings

#### Sample characteristics

All *n* = 25 participants provided demographic data. The sample was predominantly female (80%), covering different age groups and work settings. Participants from eight of Germany’s 16 federal states took part in the study, with most coming from central to northern regions, particularly the metropolitan area of Hamburg. A detailed sample description can be found in Table 1. Interviews lasted 34.4 min on average (range: 23 to 57 min). Participants cared for 33.4 patients per month on average (range: 6 to 60 patients/month) and experienced an average of 2.2 patient deaths each month (range: 0.25 to 12.5 patient deaths/month). Participants perceived a mean of 28.4% of all experienced patient deaths as distressing (range: 0 to 100%).

**Table 1:** Sample characteristics.

Characteristics	Frequency
**Age group**	
< 30	6 (24%)
31–40	5 (20%)
41–50	4 (16%)
51–60	5 (20%)
> 60	5 (20%)
**Gender**	
Female	20 (80%)
Male	5 (20%)
**Federal state** ^a^	
Hamburg	7 (28%)
Lower Saxony	5 (20%)
Bavaria	4 (16%)
North Rhine-Westphalia	3 (12%)
Saxony	2 (8%)
Saxony-Anhalt	1 (4%)
Schleswig-Holstein	1 (4%)
Baden-Wuerttemberg	1 (4%)
**Work setting** ^b^	
Outpatient psycho-oncological services at a hospital	8 (32%)
Inpatient psycho-oncological consultation service at a hospital	10 (40%)
Hospital inpatient psycho-oncology liaison service	5 (20%)
Cancer counseling center	5 (20%)
Outpatient psychotherapeutic practice	6 (24%)
Both inpatient and outpatient psycho-oncological services at a hospital	6 (24%)
Partial inpatient or inpatient palliative care	6 (24%)
Outpatient palliative care	1 (4%)
**Working experience (in years)**	
< 1	0 (0%)
1–4	9 (36%)
5–10	6 (24%)
11–15	3 (12%)
16–20	1 (4%)
> 20	6 (24%)
**Number of colleagues**	
None	3 (12%)
1–5	6 (24%)
6–10	7 (28%)
> 10	9 (26%)

^a^Federal states were excluded from the list if no participants worked in them. ^b^Multiple selection was possible.

#### Themes and subthemes

This manuscript provides an overview of key findings related to the main objectives of the study: identifying the impacts of patient deaths on psycho-oncologists, their coping strategies, and potential unmet needs. The main research questions structured the overall coding tree. Within these overarching questions, we identified major themes and three levels of subthemes, resulting in a total of 22 themes, 54 level-1 subthemes, 15 level-2 subthemes, and 15 level-3 subthemes. The complete coding tree is available in supplementary file 3.

### Impact of patient deaths on psycho-oncologists

Psycho-oncologists reported that the impact of patient deaths varied significantly across different aspects of their lives. However, they emphasized that not all patient deaths had a noticeable impact, with the level of distress influenced by multiple factors, detailed below. While all participants could recall at least one memorable case, only some indicated they sometimes felt no distress after a patient’s death: *“There are also deaths of people that I have cared for*,* where I simply say ‘Okay*,* [the patient] has also passed away.’ And where there isn’t such a big resonance [inside of me]*,* when we didn’t share that much.”* (Interview 7).

Once occuring, the impact could be categorized into three main themes: emotional and cognitive responses, effects on professional life, and effects on personal life.

### Emotional and cognitive impact of patient deaths

Participants reported being emotionally affected by patient deaths, with many experiencing significant distress. Many psycho-oncologists reported feelings of sadness or helplessness after a patient’s death, some expressed shock or a world-weariness (*Weltschmerz*). Few described feeling anger, shame or guilt following patient deaths. Adding to this range of emotions, some participating psycho-oncologists also reported feeling gratitude, relief or content as an emotional impact following patient deaths. Notably, participants distinguished between personal and professional grief, with few describing professional grief generally being less intense and shorter in duration. A recurring cognitive impact were persistent or reoccurring thoughts about deceased patients, as one participant phrased it: *“And these are the things - it’s not pain*,* I think it’s melancholy - that remain so much that the thoughts keep coming back.”* (Interview 11).

### Professional impact and quality of care

The most frequently reported professional impact was a positive one: finding a way to deal with patient deaths, as some participants put it: developing a “routine” for dealing with death, which brought a sense of composure and calmness. However, few participants noted that this acclimatization led to decreased empathy and, as in one case specifically stated, therefore could impair short-term quality of care: *“So I would say that the patient’s death had an effect on how empathetic*,* how capable I was on that day.”* (Interview 2). Overall, the professional environment also seemed to hold a few drawbacks. Some psycho-oncologists doubted the legitimacy of their grief, and few feared stigmatization, and felt limited in expressing their emotions in a professional setting. One participant phrased their questions as follows: *“And here I think I’m still asking myself the question: Is there room for [my own distress]? Is it too much? Is [being distressed] even okay?”* (Interview 4), while other participants stated clearly: *“And the only place you could go to be sad or indignant was the bathroom.”* (Interview 16) or *“At the beginning I thought that it is not professional to have a grief reaction myself [following a patient’s death].”* (Interview 10).

Additionally, some psycho-oncologists expressed doubts about their competence within their professional role due to their experiences with patient deaths. One participant illustrated his journey with anger as a part of his professional grieving process: *“But as a psycho-oncologist*,* I thought that would be wrong if I felt [anger]*,* that I had to promote this consent [towards death]*,* so to speak*,* and set an example. That’s what I thought. And I realized I couldn’t do that. I couldn’t do it at all. I was full of anger and I thought that this anger wasn’t a really professional attitude. And it actually went so far that I thought*,* if it goes on like this*,* I won’t be able to do this job.”* (Interview 18).

### Impact on personal life

Despite professional grief typically being confined to the workplace, some participants reported spill-over effects into their personal lives. This spill-over made it challenging to separate professional and personal life, especially regarding patient deaths. One participant explained: *“So the relatives are naturally confronted with a lot of suffering. But of course*,* we [the involved healthcare professionals] are too. And that’s hard to bear. And I take that home with me (…).”* (Interview 18). While few reported negative impacts, such as loss of spiritual beliefs or withdrawal from activities, most noted positive effects. These included heightened awareness of life’s fragility and a stronger alignment with personal values and beliefs. Additionally, the routine developed in dealing with frequent patient deaths seemed to help participating psycho-oncologists cope with deaths in their personal lives, providing a sense of calm: *“I’ve had to say goodbye to a lot of patients in my professional life. And that’s why it’s no longer scary. Death isn’t as scary for me as it is for people who haven’t had much to do with it (…).”* (Interview 25).

### Factors influencing the impact of patient deaths

Participants reported that their level of distress experienced was influenced by several factors. The frequency as well as the manner of the patient’s death, especially if it was painful, unexpected, or directly witnessed by the psycho-oncologist, heightened distress, as reported by most participating psycho-oncologists. One psycho-oncologist explained: “*It is also very upsetting for everyone involved [in a patient’s care] when [a patient] has not [died] peacefully (…). Instead*,* they [died] screaming or convulsing or something like that. That is perhaps also stressful and has a longer lasting effect.”* (Interview 3). The quality of the psycho-oncologist-patient relationship also played a significant role for many of them. Long-term or deep relationships, along with high levels of sympathy or dissatisfaction with the care provided, heightened levels of distress of participants. Patient characteristics, such as young age or denial of death, were described as impactful, as were the psycho-oncologists’ personal circumstances, such as similarity in age with the patient or concurrent personal crises. The method and timing of communication about a patient’s death were also reported to affect distress levels, with uncertainty about a patient’s status being particularly distressing: *“I’ll say that on the palliative care ward*,* I somehow experienced more patient deaths*,* but also [had more information regarding] when it was time [for a patient to die]*,* how [they passed away]*,* and it was easier for me to find peace with that*,* I think*,* than with these unclear cases.”* (Interview 10).

### Psycho-oncologists’ coping with patient deaths

Psycho-oncologists reported a variety of strategies for coping with patient deaths, as outlined in Table 2. These strategies were intended to facilitate successful coping, which was characterized by the psycho-oncologists’ ability to bid farewell to the deceased patient. The primary goal of coping, in this context, was to enable psycho-oncologists to *“round off”* (e.g. Interview 7 and 18) the relationship they had developed, allowing them to effectively let the patient go.

**Table 2:** Psycho-oncologists’ strategies in coping with patient deaths.

Theme	Sub-theme	Supporting quotation
Interpersonal coping strategies		
Seeking support	From colleagues (including supervision and peer consulting sessions)	“Within our team there are a lot of colleagues, who have experienced many patient deaths, and everyone has their own way of dealing with it and our team is very open and we’re taking time to listen to each other, what is on our minds. That’s what the team is there for.” (Interview 20) “We have peer consulting rounds where we talk about how we’re doing with [patient deaths], also listening to how others deal with it, how it is for them too. And I think that’s really helpful.” (Interview 11)
	From friends and family	„My husband is also an interlocutor for me, so I tell him that [a patient] made it or did not make it.” (Interview 5)
	Seeking spiritual guidance (e.g. from clergy members)	“And I went to see a colleague from the pastoral care department and she did a great job.” (Interview 1)
Affective relief (expression of emotions)		“And that, I think, is crucial, that [grief] can be expressed in some way.” (Interview 8) “It always helps me to just say out loud [that a patient died]. Then I can somehow take it more easily and I think it weighs less on me.” (Interview 9)
Contacting the deceased relatives or colleagues		“And occasionally I also contacted relatives if I had the feeling that I needed to find out more about the particular circumstances [of said patient’s death].” (Interview 3)
Saying goodbye to the deceased’s body		“But when they (…) die on the ward, I often go and stand next to [the body] and wish them a safe passage.” (Interview 11)
Intrapersonal coping strategies		
Finding the right balance	Taking a break	“I definitely paused for a moment.” (Interview 14) “And I go on lots of vacations, take lots of short trips.” (Interview 24)
	Seeking balance or distraction	„That when I go home, I don’t want to have anything more to do with work, so I just kind of watch trash TV or drink coffee or immerse myself in the newspaper and try to completely let go of this hospital world.” (Interview 18)
	Leaving the place of work	“And then when I walk home, I think about [the deceased patient] and try not to take [them] home with me.” (Interview 11)
	Seperating one’s professional and private life	“In other words, at home, so to speak, or in my private sphere, I practically left it [patient deaths] out completely.” (Interview 8)
Activities	Music	„In general, I think one way for me to deal with this is definitely music (…).” (Interview 4)
	Movement (e.g. going for a walk)	“And that, this movement, the way there, that’s really good for me.” (Interview 23)
	Nature	“That is also a great resource for me, nature and my dog.” (Interview 5)
	Lighting a candle	“So what helps me is to process [a patient’s death] is (…) to light a candle as a ritual, either at work or at home if it doesn’t fit in here.” (Interview 10)
	Visiting a spiritual space (e.g. chapel, church, cemetary etc.)	“Then I go back to our chapel and light a candle.” (Interview 2)
	Closing the patient file	“It’s also a kind of ritual for me to close a patient’s file, to make a note in the documentation that the patient has died.” (Interview 3)
Adjusting one’s stance	Acceptance	“I think that’s always a development that you go through [… to] learn to accept that dying is part of life.” (Interview 13)
	Relying on one’s beliefs	„So I would say that is a necessary prerequisite for me to be able to do this work at all, to have dealt with what death and dying mean to me, how I myself might wish for it, imagine it and always think about how I would want to deal with it if I were in such a situation.” (Interview 3)

Coping strategies were categorized into interpersonal and intrapersonal categories. Interpersonal strategies involved interaction with others, while intrapersonal strategies were more self-directed. A third significant category that emerged was rituals, which could be either interpersonal, intrapersonal, or both. Rituals appeared to hold a special significance, as they often encapsulated other coping strategies or a combination of them, performed in a repetitive and somewhat formalized manner. Therefore, the significance of a ritual did not lie with its specific coping mechanism, e.g. lighting a candle or reading all deceased patients’ names each week in a team-meeting, a ritual’s significance seemed to be the reliable framework it provides to cope with patient deaths. Paying tribute to the deceased seemed to be an important part of rituals. One participant stated: *“So what I really do with everyone*,* I sit down and do a farewell ritual by myself. I then take the patient file and think again about what kind of person they were and then [close the deceased patient’s case formally]. That’s a very important ritual for me*,* that I sort of say goodbye for myself.“* (Interview 21).

Barriers to successful coping were identified, including time pressure and the perceived need to adhere to certain implicit protocols of behavior after a patient’s death. One such perceived expectation was to maintain strict work-life boundaries when coping with patient deaths. For example, one participant (Interview 22) remarked: *“This typical ‘you’re not allowed to take work home [referring to a professional grieving experience]’, I think that’s nonsense.”* However, these perceived protocols were highly subjective and varied greatly among participants, making it challenging to identify common themes.

Additionally, some psycho-oncologists noted that suppressing their own emotions, such as avoiding situations that might trigger emotional responses, or being unable to express emotions, possibly due to time constraints or a lack of collegial support, hindered the process of letting go of a patient.

### Psycho-oncologists’ coping support needs

Psycho-oncologists’ potentially unmet needs in coping with patient deaths were explored as well. While some participants felt adequately equipped to handle patient deaths in their careers and did not perceive any unmet needs, many interviewees addressed areas for potential improvement.

Many psycho-oncologists highlighted a need for dedicated spaces where they could discuss their experiences with patient deaths and the emotional and overall impact these events have on them, as this psycho-oncologist reported: *“I’m more interested in creating a space where this [talking about deceased patients] can take place instead of going back to business as usual.”* (Interview 15). For some, there was a desire for additional opportunities to explore their own challenges in coping with death, such as through self-awareness seminars. Some participants also expressed a preference for sharing their distress in a more private setting, such as individual supervision sessions.

Moreover, some psycho-oncologists indicated a desire for more team-based supervision sessions and the implementation of team rituals to collectively grieve deceased patients. One participant explained this desire as follows: *“So I think that [grief] is an important team topic because I think that’s where we have the greatest resource*,* to somehow find a way of dealing with [patient deaths]*,* perhaps collective rituals (…)*,* perhaps to improve something.”* (Interview 1).

A common area for improvement was related to communication pathways within institutions regarding patient deaths, with a need to inform all HCPs involved in the care of a patient about this patient’s death in a personal and timely manner, as highlighted by one participant: *“You [need to] find out promptly when someone has died. Currently you might not get the information at all or only by chance or with a long delay.”* (Interview 15).

Finally, many psycho-oncologists reported unmet informational and educational needs, as highlighted by participant 16, responding to the question whether they wished for continued education in coping with patient deaths: *“That would be great*,* wouldn’t it?”*. Although most participants had received some form of educational preparation for coping with patient deaths during their professional training, this training was typically not part of their university curriculum, but was instead acquired through postgraduate training initiatives.

## Discussion

Patient deaths are not uniformly distressing to psycho-oncologists, but some significantly impact both their professional and personal well-being. Factors such as the expectedness of a patient’s death and the depth of the shared relationship seem to influence the level of distress experienced by psycho-oncologists following patient deaths. Once it manifests, participants reported that the impact extends beyond the professional domain into private life, sometimes leading to positive outcomes like perspective-taking and aligning life with personal priorities. However, the described spill-over into personal life and the difficulty in maintaining professional boundaries are complex and multifaceted issues. While healthy boundaries are crucial for the well-being of healthcare professionals^22–24^ it is important to acknowledge that professional grief may not always be fully contained within the work setting. Grieving is a highly individual process, and it should be recognized that expressing grief in one’s private life, when necessary, is not inherently problematic. Few participants noted that feeling pressured to contain grief solely within the professional setting hindered their ability to cope effectively with patient deaths. Therefore, psycho-oncologists should not feel compelled to leave their emotions strictly at work, as this may counteract healthy coping.

Overall, while individuals should feel allowed to grieve in any way and place that suits them personally, blurred boundaries between psycho-oncologist’s professional and private life may increase the risk of negative outcomes associated with professional grief. Hence, there is a delicate balance between empowering healthcare professionals to grieve and address their emotions within the workplace and avoiding the imposition of overly rigid expectations that professional emotions must be solely dealt with in that context. Institutions should at least provide options for psycho-oncologists to grieve deceased patients within the workplace to support healthy boundary maintenance. Regardless of where HCPs choose to process their grief, employers should take responsibility for creating a work environment conducive to coping and offer designated spaces for professional grief, allowing individuals to choose whether they grieve at work or in their private life.

Various coping strategies were identified, with rituals—whether personal, team-based, or institutionally supported—playing a central role in acknowledging and “rounding off” the patient relationships. Social support, particularly from colleagues, was seen as crucial for effectively coping with patient deaths.

This study sheds light on the impact of patient deaths on psycho-oncologists, an often overlooked group of HCPs in this field of research^5,6^. Patient deaths seem to affect psycho-oncologists similarly to other HCPs, such as nurses or physicians, often leading to symptoms of professional grief like sadness or helplessness^6,13,16,25,30^. This is an important finding, as the nature of the care delivered by psycho-oncologists varies from medical and nursing care^31,32^. However, one notable distinction identified in our study is that psycho-oncologists did not commonly report feelings of guilt following patient deaths. This may be attributable to said differences in professional roles associated with the differing nature of care. This study provides preliminary evidence that HCPs, no matter how different their roles within the health care system, may be equally affected by professional grief. While professional grief may first and foremost be a natural and adaptive response to patient loss, it can also lead to negative outcomes, such as feelings of sadness and helplessness negatively influencing psycho-oncologists’ well-being, as well as temporarily decreased empathy, impairing care quality^33,37^. However, psycho-oncologists reported various strategies to cope with patient deaths successfully, which may help to prevent these negative effects from arising.

Additionally, this study contributes to the corpus of research identifying professional grief as a distinguishable phenomenon from general grief^5,6,34^. Participants differentiated their experiences with patient loss from the loss of loved ones, yet spill-over effects were noted. Emotionally, professional grief appears to resemble general grief, as similar emotions are felt; however, it is typically experienced as less intense and of shorter duration. Yet, participants differentiated the two experiences and stated challenges specifically associated with, for example, their professional role within professional grief. This interplay suggests that, although professional grief and general grief may be distinct, they likely influence each other and professional grieving experiences might even ease distress felt when grief occurs in a private setting due to acclimatization and routine in coping with deaths. Therefore, while grief may serve as an appropriate ‘umbrella term’, professional grief appears to be at least a specific form of grief, warranting further research.

### Implications

This study highlights both the positive and negative impacts of patient deaths on psycho-oncologists. Similar to nurses and physicians, psycho-oncologists experience symptoms of professional grief, though feelings of guilt and responsibility for patient outcomes appear less central^34,38^ likely due to differing professional roles. While professional grief varies across healthcare professions^6,29^ identifying predictors of its occurrence remains crucial.

Understanding the symptoms, prevalence, and intensity of professional grief among different HCP groups is essential to distinguish adaptive responses from psychological burdens that may affect well-being and care quality^5,8,39^. Moreover, exploring the specific emotional profile of professional grief—particularly how it compares to general grief and the targets of emotions such as anger—warrants further investigation. Future studies could benefit from examining these nuanced differences to gain a more comprehensive understanding of professional grief.

More research is needed to explore professional grief among psychosocial HCPs, especially outside North America, given that previous studies primarily focus on North American and Israeli healthcare contexts. Although professional grief, much like grief in general, may represent a cross-cultural human response to patient loss, further studies in diverse cultural and healthcare settings are essential to confirm or challenge this notion. While intercultural differences were not a primary focus of this study, only preliminary observations can be made regarding cultural variations in professional grief. Granek et al.^25^ suggest that the emotional experience of professional grief (e.g., sadness, helplessness, and guilt) is largely cross-cultural, which aligns with the present findings. In the sample of German psycho-oncologists, guilt was not commonly reported—a difference potentially attributable to professional roles rather than cultural factors. Previous studies also indicate that religious and historical contexts may shape professional grief. However, it remains unclear how culture, professional roles, and other factors—and their interplay—affect the experience and expression of professional grief. Despite these observations, there is no conclusive evidence of substantial cultural differences, and professional grief, like grief in general, appears to be a fundamentally human experience that transcends cultural boundaries.

Additionally, our findings suggest that healthcare institutions should support coping strategies, especially peer-support, to mitigate the negative impact of patient deaths, enhancing psycho-oncologists’ well-being and care quality. Developing and evaluating targeted interventions, peer support, and comprehensive education can help psycho-oncologists manage the emotional challenges related to patient deaths. There remains an unmet need for educational resources and self-awareness workshops to better prepare psycho-oncologists in coping with their patients’ deaths.

### Strengths and limitations

To our knowledge, this study is the first to explore experiences with patient deaths and professional grief among psycho-oncologists within European healthcare contexts. Our data analysis involved two team members, one of whom was not involved in data collection. Additionally, data was coded following a thorough and pre-agreed quality control process and COREQ reporting guidelines were adhered to.

However, there are several limitations to our study. Despite our efforts to purposefully sample participants varying in potentially influencial characteristics such as years of experience in psycho-oncological care, specific work-setting and location of employment, our sample is somewhat dominated by psycho-oncologists from the metropolitan area of Hamburg. Additionally, no psycho-oncologists working in rehabilitation clinics, a common setting in Germany, participated in this study. This limitation may be less significant since cancer patients fit for rehabilitation are typically not in an acute palliative state and are less likely to die at that point of care. Nevertheless, our sample remains a convenience sample. Additionally, there may be a self-selection bias. This could result in a somewhat positive portrayal of psycho-oncologists experiences and ability to cope with patient deaths, as psycho-oncologists who struggle significantly may have been hesitant to participate due to stigmatization and potential feelings of shame. Consequently, while generalizability is often not the aim in qualitative studies^40^ it remains a concern in this case.

Furthermore, we did not return the interview transcripts to participants for feedback, as we deemed this process too effortful for the participants. Although we strived to ensure accurate transcription by involving two researchers, we acknowledge that not seeking participant validation may still represent a limitation.

Additionally, conducting telephone interviews on such a sensitive topic may itself represent a limitation. While this approach allowed for national accessibility and minimized logistical challenges, it also limited the ability to observe non-verbal cues, which could have enriched the data.

Another limitation relates to the gender distribution within our sample, which was predominantly female. While this reflects the gender ratio within the field of psycho-oncology, it might have influenced our results, especially given that some studies suggest that professional grief may differ by gender^27,29,41^.

Finally, our study did not assess how the therapeutic relationship building before the patient’s death might influence professional grief. This should be assessed in future studies, as it could be helpful to shape future continuing education.

## Conclusion

We highlighted both positive and negative impacts of patient deaths and identified several characteristics of professional grief among psycho-oncologists. Additionally, we identified key coping strategies that institutions could support to help mitigate the negative effects, thereby improving psycho-oncologists’ quality of life and potentially the quality of care they provide. This study serves as a foundation for further research and the development of targeted interventions to address professional grief and meet the needs of psychosocial HCPs involved in cancer care. Further research is needed to explore professional grief in depth and tailor support strategies to specific professions and healthcare contexts. To support psycho-oncologists, institutions should facilitate ways for them to achieve a “rounded off” farewell to deceased patients. Rituals and peer support have been identified as valuable coping strategies, suggesting that ritualized peer support could be an effective approach.

## Electronic supplementary material

Below is the link to the electronic supplementary material.

Supplementary Material 1

Supplementary Material 2

Supplementary Material 3

## Data Availability

The datasets used and/or analysed during the current study are available from the corresponding author on reasonable request.
