# Supplementary material for: A qualitative interview study on psycho-oncologists’ experiences with patient deaths in Germany
Source: Sci Rep. 2025 Jul 1;15:22328. doi: 10.1038/s41598-025-06991-x (PMC12216660; doi:10.1038/s41598-025-06991-x)
Supplement: Supplementary file 1 — Supplementary Material 1 [file 41598_2025_6991_MOESM1_ESM.docx]

**Supplementary file 2: Guideline for semi-structured interviews**

| **Verbal consent** | We will now proceed and start our interview. I will switch on the recording devices.  *(SW switches on devices)*  I have now started the audio recording, is that okay with you?  *(Verbal consent?)*  Then let's start now. |
| --- | --- |
| **Subject** | **Probing questions** |
| Introduction  Sharing own experiences (leading over to the experience of professional grief) | In our interview today, I would like to ask you about your personal experiences with the death of patients in psycho-oncology. It will also be about how you deal with it. There is no right or wrong here; I am interested in your individual views and experiences.  I would like to ask you to remember a case in which a cancer patient you accompanied died. If you like, you can tell me briefly about this case. |
| Impact of patient deaths | 1. How did the death you just described to me, or other patient deaths, affect you?    1. Ask if necessary: Were there any professional or private effects?    2. Ask if necessary: Can you describe these effects to me in more detail? 2. Do you have any personal beliefs or convictions in connection with death?    1. Ask if necessary: Have these changed in the course of your experiences with the death of patients? 3. To what extent does your experience with the death of this patient or of patients in general differ from your experience with private deaths?    1. Ask if necessary: Are there any similarities between the two experiences?    2. Ask if necessary: Are there any differences?   Optional questions:  If you had to give a name to your reactions to the death of a patient, what would you call them? |
| Coping mechanisms for professional grief | 1. How did you cope with the death of the patient you just told me about, or with the death of other patients?    1. Ask if necessary: What strategies do you use to process these experiences? 2. Which of these strategies do you find helpful in dealing with the death of patients? Are there any that are not helpful for you?   Optional questions:  What strategies do you use immediately after learning of a patient's death?  Which strategies are helpful for dealing with the death of patients in the long term, i.e. weeks or months afterwards? |
| Support needs in coping with patient deaths | 1. Where do you find support for coping with the death of patients when you need it?    1. Ask if necessary: Are there offers for support in your professional environment? 2. What (additional) support would you like? |
| (Educational) Preparation for coping with patient deaths | 1. Were you prepared for coping with the death of patients during the course of your professional training?    1. If yes, could you describe what this preparation looked like? 2. Would you like ongoing or further training opportunities in dealing with the death of patients?    1. Optional question: In what context would you prefer such training? |
| **Ending of the interview**  Prioritizing of important aspects | At the end, I would like to ask you one more questions.   1. What advice would you give to new psycho-oncologists who are confronted with the death of a patient?   Finally, I would like to know if there is anything else you would like to tell me in regard to your experiences with patient deaths that I may not have asked you before? |
