# Supplementary material for: A qualitative interview study on psycho-oncologists’ experiences with patient deaths in Germany
Source: Sci Rep. 2025 Jul 1;15:22328. doi: 10.1038/s41598-025-06991-x (PMC12216660; doi:10.1038/s41598-025-06991-x)
Supplement: Supplementary file 2 — Supplementary Material 2 [file 41598_2025_6991_MOESM2_ESM.docx]

**Supplementary file 3, Coding tree**

| Theme | Subtheme (Level 1) | Subtheme (Level 2) | Subtheme (Level 3) | Description |
| --- | --- | --- | --- | --- |
| Research question 1: How do patient deaths affect psycho-oncologists? | | | | |
| Few/ no effects |  |  |  | Participants report no or few noticeable effects from one or more patient deaths; can be coded alongside emotions and/or other effects. |
| Being touched |  |  |  | Participant reports that a patient’s death/patient deaths evoke emotions; affects them emotionally, without a negative connotation. |
| Being distressed |  |  |  | Participant reports emotional distress caused by a patient’s death/patient deaths; corresponds to a negative connoted version of “being touched”. |
| Doubts about the legitimacy of own emotions/  distress |  |  |  | Participants question the extent to which their own (emotional) concern/reactions are appropriate; indicates disenfranchised grief. |
|  | Limited space for own emotions |  |  | Participant reports feeling as if his/her own (emotional) reaction (grief) has more space or is (societally perceived) as more appropriate in regard to deaths occurring in the private sphere. |
|  | Fear of stigmatization |  |  | Participant reports fears of stigmatization, e.g., related to own experiences of racism or general professionalism. |
| Factors contributing to higher/lower distress following patient deaths |  |  |  | “Why do clinicians find some deaths more distressing than others?" |
|  | Related to the nature of the death/manner of dying |  |  | Participant reports heightened distress due to direct presence at the moment of death, "suffering deaths" (patients experiencing pain or lack of symptom control), unexpected deaths, or a high frequency/density of patient deaths. |
|  | Related to the therapeutic-supportive relationship |  |  | Participants report increased distress when clinicians are dissatisfied with their care (also due to external factors like inability to control symptoms), strong sympathy between clinician and patient, or long-term and deep relationships. |
|  | Related to patient characteristics |  |  | Participant reports increased distress when patients do not accept/deny their impending death or when patients are young (especially children). |
|  | Related to the psycho-oncologist's personal life or characteristics |  |  | Participant reports high identification with patients (e.g., similar age, professional proximity, shared migration background), death of a loved one, or other crises/ an overall reduced capacity for resilience or emotional stability. |
|  | Related to the communication of patient deaths |  |  | Participant reports increased distress when the death is uncertain (no definitive notification that the patient has died), or communication of patient deaths is delayed and/or "impersonal" (e.g., through patient file/ digital documentation systems etc.). |
| Emotions |  |  |  |  |
|  | Content |  |  | Participant reports feelings of content, e.g. with own work, quality of provided care. |
|  | Relief |  |  | Participant reports feeling relieved, e.g. because the deceased patient’s suffering came to an end due to death. |
|  | Gratefulness |  |  | Participant reports feeling grateful, e.g. for shared relationship, trust and intimacy with the deceased patient. |
|  | World-weariness (“Weltschmerz”) |  |  | Participant reports feeling somewhat weary about e.g. life being unfair. |
|  | Helplessness |  |  | Participant reports feeling helpless, overwhelmed, left alone in some sort; “resources do not match tasks”. |
|  | Sadness |  |  | Participant reports feeling sad. |
|  | Shock |  |  | Participant reports feeling surprised, taken off-guard, shocked by patient death. |
|  | Anger |  |  | Participant reports feelings of anger, e.g. towards the deceased patient, the circumstances under which care had to be provided (component of moral distress), etc. |
|  | Guilt |  |  | Participant reports feeling guilty. |
|  | Shame |  |  | Participant reports feelings of shame surrounding patient death; e.g. due to own distress. |
|  | Grief |  |  | Participant reports feeling grief about a patient’s death. |
| Cognitive „symptoms“ |  |  |  |  |
|  | Reoccuring thoughts about patient(s) |  |  | Participant reports lasting or reoccuring thoughts about deceased patient(s). |
| Impact on professional life/performance |  |  |  |  |
|  | Impaired ability to empathize with patients |  |  | Participant reports feeling less able to empathize with patients due to confrontation with patient deaths. |
|  | Doubts about professional competence |  |  | Participant reports doubting his/her own professionalism/ overall competence as a psycho-oncologist; feelings of inadequacy. |
|  | Routine in dealing with patient deaths |  |  | Participant reports developing/having developed a routine in experiencing and coping with patient deaths; frequent confrontation leads to familiarization, which aids in keeping composure. |
| Impact on personal life |  |  |  | Spill-over; effects that present in personal life as well (might be present in professional life too/”spilled-over” from professional into private domain. |
|  | Withdrawing from leisure activities / loss of interests |  |  | Participants report withdrawing from activities or interests previously done for pleasure/ seen as resources. |
|  | Changes in beliefs concerning death |  |  |  |
|  |  | Dissolution/loss of previous beliefs |  | Participant reports loss or dissolution of (religious) beliefs; does not have to be subjectively seen as negative. |
|  |  | Consolidation/clarification of own beliefs |  | Participant reports changes in beliefs but evaluates changes clearly as positive. |
|  | Serenity in dealing with death and dying |  |  | Participant reports frequent confrontation with death/dying in professional life lead to increased serenity/composure in dealing with death of loved ones. |
|  | Difficulties separating private and professional life |  |  | Participant reports difficulties in keeping boundaries between professional and private life/ maintaining a work life balance; participant does not have to evaluate the “lack of boundaries” as negative. |
|  | Clarifying life values/ giving perspective |  |  | Participant reports exposure to patient deaths aided them in e.g. setting personal priorities, making decisions that are right for oneself, living life the way that is right for one personally (in the knowledge that life itself is fleeting). |
| Differences between professional grief and grief in general |  |  |  |  |
|  | Difference in the way of saying goodbye |  |  | Participant reports differences in the way they say goodbye to the deceased; e.g. attending funerals is appropriate within the private domain, but less appropriate or inappropriate concerning deceased patients. |
|  | Difference in grief’s duration |  |  | Participant reports professional grief differs in duration (taking less time). |
|  | Difference in felt intensity |  |  |  |
|  |  | Due to increased regulatory capacity in the event of patient death |  | Participant reports self-regulation and emotion regulation are easier regarding patient deaths; might be due to higher demands on one's own ability to regulate (professionalism). |
|  |  | Due to differences in the relationship’s depth |  | Participant reports differences in intensity due sharing a „less close bond“with patients, private relationships by nature being deeper and therefore more distressing in the event of loss. |
|  |  | Due to grief being confined to professional role |  | Participant reports with personal losses the person as a whole is emotionally shaken, the loss is fundamental, whereas professional grief “only” affects the professional role. |
| Research question 2: How do psycho-oncologists cope with patient deaths? | | | | |
| Strategies aiding successful coping |  |  |  | Strategies that aid in „rounding off” the relationship; help psycho-oncologists to “let go of the patient”. |
|  | Intrapersonal strategies |  |  | “no other person needed” |
|  |  | Activities |  |  |
|  |  |  | Nature | Participant reports using nature/natural environment as a resource, actively pursuing nature. |
|  |  |  | Music | Participant reports listening to music or playing an instrument to cope with patient deaths. |
|  |  |  | Close patient file | Participant reports closing a patient’s file as a coping strategy. |
|  |  |  | Lighting a candle | Participant reports lighting a candle as a coping strategy. |
|  |  |  | Visiting a spiritual space | Participant reports visiting a spiritual space to cope with patient deaths, e.g. a cemetary, chapel etc. |
|  |  |  | Movement | Participant reports using movement to cope with patient deaths, e.g. going for a walk or doing sports, yoga etc. |
|  |  | Adjusting one’s stance |  |  |
|  |  |  | Relying on one’s beliefs | Participant reports relying on established beliefs to reframe patient deaths; accepting death as a part of life, believing in that aids him/her. |
|  |  |  | Acceptance | Participant reports using acceptance aids him/her in coping with patient deaths. |
|  |  | Finding the right balance |  |  |
|  |  |  | Taking a break | Participant reports taking either small breaks/moments to themselves to cope with patient deaths and/or using time off of work and vacations to cope. |
|  |  |  | Seeking balance or distraction | Participant reports distracting himself/herself or explicitely doing leisure “light” leisure activities to balance out “heavy” work-related events as coping strategy. |
|  |  |  | Leaving the place of work | Participant reports leaving the place of work, having “a way home” as a coping strategy; distance between work and home life as helpful. |
|  |  |  | Seperating professional and private life | Participant reports maintaining boundaries between work and private life as coping strategy. |
|  | Interpersonal strategies |  |  | Other people have to be present in some capacity. |
|  |  | Social Support |  |  |
|  |  |  | Through colleagues | Participant reports peer support as coping strategy; informal or formalized (through supervision or peer counselling groups). |
|  |  |  | Through friends and family | Participant reports sharing a patient’s death with partners, friends or family as a coping strategy. |
|  |  |  | Through spiritual assistance | Participant reports seeking spiritual guidance or talking to e.g. clergy as a coping strategy. |
|  |  | Affective relief |  | Participant reports showing and/or sharing own emotions helps him/her in coping with patient deaths. |
|  |  | Saying goodbye to the deceased’s body |  | Participant reports (re)visiting the body of the deceased aids them in coping. |
|  |  | Contacting the deceased relatives or colleagues |  | Participant reports contacting e.g. other HCPs involved in care or the deceased’s relatives helps him/her in coping with the patient’s death; e.g. through “filling in knowledge gaps”. |
|  | Rituals |  |  | Any coping strategy performed in a ritualized manner, usually repetitive. |
|  |  | Paying tribute to the deceased |  | Participant reports paying tribute in some way as important, a component of individually chosen ritual. |
|  |  | Team or institutional rituals |  | Participant reports taking part in team or institutional bereavement rituals, e.g. patient memorials; NOT private memorials “organized” by patient’s relatives. |
| Barriers in coping with patient deaths |  |  |  | Everything hindering psycho-oncologists in successfully coping with patient deaths/ “rounding off” the relationship. |
|  | Expression of grief is impossible |  |  | Participant reports expression of own emotions, distress or grief is or feels impossible, e.g. due to interlocutors being inexistent or unavailable. |
|  | Time constraints |  |  | Participant reports feeling like he/she does not have enough time to feel and express their own emotions or to say goodbye, e.g. due to hectic schedule, other task’s urgency etc. |
|  | Pressure/perfectionism in coping with patient deaths |  |  | Participant reports own expectation or feeling pressured by other’s expectations; that there is one right way to deal with the death of patients. |
|  | Suppression of emotions |  |  | Participant reports “not wanting to feel” his/her own emotions/grief, e.g. avoiding situations that “trigger” grief or cognitively suppressing one's own emotions. |
| Research question 3: Were psycho-oncologists educationally prepared to cope with patient deaths? And if so: where? | | | | |
| YES, pre-graduate education |  |  |  | Participant reports being prepared to cope with patient deaths in university. |
|  | Psychology |  |  | Participant reports preparation as part of psychology degree. |
| YES, post-graduate training |  |  |  | Participant reports being prepared to cope with patient deaths in post-graduate training. |
|  | Psycho-oncology |  |  | Participant reports being prepared to cope with patient deaths in post-graduate psycho-oncological training. |
|  | Palliative psychology |  |  | Participant reports being prepared to cope with patient deaths in post-graduate palliative psychology training. |
|  | Systemic counseling |  |  | Participant reports being prepared to cope with patient deaths in post-graduate training to become a systemic counselor. |
|  | Psychotherapy |  |  | Participant reports being prepared to cope with patient deaths in post-graduate training to become a psychotherapist. |
| NO, pre-graduate training |  |  |  | Participant reports not being prepared to cope with patient deaths or feeling like preparation was insufficient within education in university. |
|  | Psychology |  |  | Participant reports not being prepared to cope with patient deaths or feeling like preparation was insufficient within his/her psychology degree. |
|  | Medicine |  |  | Participant reports not being prepared to cope with patient deaths or feeling like preparation was insufficient within his/her medicine degree. |
| NO, post-graduate training |  |  |  | Participant reports not being prepared to cope with patient deaths or feeling like preparation was insufficient within post-graduate training. |
|  | Psycho-oncology |  |  | Participant reports not being prepared to cope with patient deaths or feeling like preparation was insufficient within post-graduate psycho-oncological training. |
|  | Psychotherapy |  |  | Participant reports not being prepared to cope with patient deaths or feeling like preparation was insufficient within post-graduate training to become a psychotherapist. |
| Research question 4: Do psycho-oncologists have any unmet needs in regard to their coping with patient deaths? | | | | |
| No unmet needs |  |  |  | Participant reports personally not having any unmet needs in regard to patient deaths. |
| Space to discuss death and own emotional distress |  |  |  | Participant reports wishing for more space to discuss death and share own emotions in regard to patient deaths. |
| Within the institution |  |  |  |  |
|  | Improved communication |  |  | Participant reports wishing that patient deaths are communicated in a more personal and timely manner to all HCPs involved. |
| Within a team |  |  |  |  |
|  | Supervision |  |  | Participant wished for more (frequent) team supervision. |
|  | Team-based rituals |  |  | Participant shared a desire to take part in team-based rituals to say goodbye to deceased patients. |
| Individually |  |  |  |  |
|  | One-on-one conversations |  |  | Participant shared a need for more opportunities to have one-on-one conversations concerning his/her distress with patient deaths. |
|  |  | Being proactively approached |  | Participant wished for supervisors to proactively approach employees and offer conversations surrounding professional grief. |
|  | Self-awareness workshops |  |  | Participant reported wishing for more opportunities to practice self-awareness. |
|  | One-on-one supervision |  |  | Participant reported needing more opportunities for supervision, WITHOUT other team members present. |
| Educational and informational needs |  |  |  | Participant reports needing additional education and/or information surrounding patient deaths, coping with them etc. |
|  | Death/ bereavement workshops |  |  | Participant wished for workshops surrounding death or bereavement. |
